# Influences of Long-Term Exercise and High-Fat Diet on Age-Related Telomere Shortening in Rats

**DOI:** 10.3390/cells11101605

**Published:** 2022-05-10

**Authors:** Maria Donatella Semeraro, Gunter Almer, Wilfried Renner, Hans-Jürgen Gruber, Markus Herrmann

**Affiliations:** Clinical Institute of Medical and Chemical Laboratory Diagnostics (CIMCL), Medical University of Graz, 8036 Graz, Austria; maria.semeraro@medunigraz.at (M.D.S.); gunter.almer@medunigraz.at (G.A.); wilfried.renner@medunigraz.at (W.R.); markus.herrmann@medunigraz.at (M.H.)

**Keywords:** telomeres, telomerase, shelterin, moderate exercise, high-fat diet, Sprague Dawley rats

## Abstract

(1) Obesity and exercise are believed to modify age-related telomere shortening by regulating telomerase and shelterins. Existing studies are inconsistent and limited to peripheral blood mononuclear cells (PBMCs) and selected solid tissues. (2) Female Sprague Dawley (SD) rats received either standard diet (ND) or high-fat diet (HFD). For 10 months, half of the animals from both diet groups performed 30 min running at 30 cm/s on five consecutive days followed by two days of rest (exeND, exeHFD). The remaining animals served as sedentary controls (coND, coHFD). Relative telomere length (RTL) and mRNA expression of telomerase (TERT) and the shelterins TERF-1 and TERF-2 were mapped in PBMCs and nine solid tissues. (3) At study end, coND and coHFD animals showed comparable RTL in most tissues with no systematic differences in TERT, TERF-1 and TERF-2 expression. Only visceral fat of coHFD animals showed reduced RTL and lower expression of TERT, TERF-1 and TERF-2. Exercise had heterogeneous effects on RTL in exeND and exeHFD animals with longer telomeres in aorta and large intestine, but shorter telomeres in PBMCs and liver. Telomere-regulating genes showed inconsistent expression patterns. (4) In conclusion, regular exercise or HFD cannot systematically modify RTL by regulating the expression of telomerase and shelterins.

## 1. Introduction

The shortening of telomeres, protective nucleoprotein structures at the end of all chromosomes, is a hallmark of aging that compromises genomic integrity and alters the expression of many genes [1]. Due to the inability of the DNA polymerase to fully replicate the 3′ end of chromosomes, telomeres progressively shorten with every cell division until they reach a critical threshold below which they lose their DNA-protecting properties and send cells into senescence or apoptosis [2]. Numerous studies have shown that short and dysfunctional telomeres are linked to premature atherosclerosis, diabetes, and hypertension [3,4,5,6,7]. Furthermore, telomere length is inversely related to mortality risk [8,9,10,11].

Aging is an individual process that can be influenced by modifiable lifestyle factors, such as physical activity, nutrition, stress, sleep, smoking and others [12,13,14,15,16,17,18,19,20,21]. Physical inactivity and obesity are established triggers of metabolic dysfunction, chronic inflammation, and oxidative stress, which increase the risk of atherosclerosis, diabetes, hypertension, dementia, and other age-related diseases [22,23]. Based on previous studies, it has been speculated that physical inactivity and obesity also accelerate telomere attrition and promote telomeric dysfunction [24]. Conversely, it has been proposed that regular exercise and a balanced diet promote healthy aging not only through beneficial effects on body composition, metabolic function, vascular function, blood pressure, inflammatory processes, and mental stress [25], but also through the preservation of telomere length and function [13,26,27,28,29,30]. It has further been hypothesized that lifestyle-induced effects on telomeres are mediated through telomere-regulating proteins, such as telomerase and shelterins [16,18,19,21,27,31]. Telomerase can counteract telomere shortening by adding new hexanucleotides to the telomeric ends. With the help of shelterins, a complex of six individual proteins, telomeres assume a unique three-dimensional structure that is essential for their function. Upon binding of the shelterin complex to the TTAGGG motif, telomeric DNA folds backward forming a structure known as t-loop [32,33]. A breakdown of the t-loop structure, called telomere uncapping, represents a critical mechanism that promotes age-related vascular dysfunctions, cellular senescence and inflammation beyond telomere shorting [34].

Obesity and physical inactivity are highly prevalent in modern societies [35]. According to the World Health Organization (WHO), approximately 30% of the global population is obese [36] and the Centre of Disease Control in the US has estimated an overall prevalence of physical inactivity in the US of approximately 25% [37]. Despite intensive research activities, the mechanisms that mediate the increased risk of chronic degenerative diseases in obese and inactive individuals are incompletely understood. Previous studies have nurtured the idea that both of these lifestyle factors could increase disease risk and mortality through an enhancement of telomere shortening that compromises genomic integrity [38]. However, the results of observational studies are controversial, and experimental evidence that establishes a mechanistic link between obesity, physical inactivity and accelerated telomere shortening is largely lacking. Several observational studies showed an inverse relationship between telomere length (TL) in leucocytes (LTL) and BMI [39,40], whereas others found the opposite [41] or no significant association [42,43]. Inverse correlations have also been reported for LTL and different indices of body composition, such as body fat content, waist circumference, waist-to-hip ratio, and nuchal fat thickness [43,44,45,46,47,48,49,50]. In contrast, two mouse models of obesity and metabolic syndrome failed to show accelerated telomere shortening despite an upregulation of telomerase and senescence-associated genes, such as checkpoint kinase 2 (*Chk2*), *p53*, and *p21* [22,23].

Considering that exercise is a highly cost-effective way to improve health and to prolong life [51,52,53,54,55], obese individuals are often prescribed a physical activity program with moderate endurance exercise, such as walking or cycling [56]. Observational studies have reported higher LTL in exercising individuals of different age groups and different activity levels [15,17,18,57,58]. However, available prospective and interventional studies provide conflicting results. In a 5-year longitudinal study by Soares-Miranda L et al., physical activity and physical performance were unrelated to LTL [59]. In contrast, Werner et al., showed increased LTL and an upregulation of telomerase and telomere repeat binding factor (TRF) 2 after 6 months of aerobic endurance training or high intensity training [17,18]. The results from animal studies are also inconclusive. While Ludlow et al. showed a preservation of TL in cardiomyocytes and hepatocytes of exercising mice [16,17,21,60], Werner et al. did not find differences between cardiac TL of exercising and sedentary mice [16,17,21,60]. Regardless of potential effects on TL, exercise seems to alter the expression telomere-regulating proteins, such as telomerase and shelterins [60].

Whether or not obesity and physical activity are causally related to telomere length and the expression of telomere-regulating proteins remains elusive. Furthermore, previous studies are limited to analyses of TL in leucocytes, myocardium, skeletal muscle, and liver. Additionally, potential interactions between the consumption of a hypercaloric diet and regular exercise has not been studied systematically. This aspect is of particular relevance as exercise is often used to compensate bad eating habits and to treat obesity. Therefore, the present study aimed to address this gap of knowledge by mapping TL in leucocytes and 9 solid tissues from aged sedentary rats that were fed for 10 months either a normal chow-based diet (ND) or a synthetic high-fat diet (HFD). In order to explore potential interactions between the consumption of HFD and exercise, half of the animals from both groups performed regular treadmill running with moderate intensity.

## 2. Materials and Methods

### 2.1. Animal Model

Ninety-six female Sprague Dawley (SD) rats were purchased from Janvier Labs (Le Genest-Saint-Isle, France) at four months of age. The animals were kept in groups of three animals per cage under constant conditions on a 12 h/12 h light/dark cycle at the core facility animal housing at the Medical University of Graz (Austria). Temperature was maintained between 22 and 25°C. Humidity ranged between 55 to 58%. After one week of acclimatization, the animals were randomly assigned to receive either a standard diet (ND) (Altromin, Lage, Germany) with 3230 kcal/kg and 11% fat or a custom-designed beef-tallow high-fat diet (HFD), rich in saturated fatty acids (SFA), in particular C16:0 and C18:0, with 5150 kcal/kg and 60% fat (Table 1; ssniff, Soest, Germany). Food and tap water were provided ad libitum. 

### 2.2. Experimental Design and Treatment 

Animals were randomly allocated to following 4 groups, each consisting of 24 animals: coND, exeND, coHFD and exeHFD. coND and exeND animals were fed with ND for the entire study period, whereas coHFD and exeHFD received HFD. The animals from exeND and exeHFD groups performed a 10-month exercise program consisting of 30 min of forced running on a treadmill (Panlab, Barcelona, Spain) on five consecutive days followed by 2 days of rest. The running speed was constant and set at 30 cm/s. The training protocol was based on previous experimental studies [61,62,63,64]. The animals in the coND and coHFD groups did not exercise and had no access to a running wheel. These animals were used as sedentary controls.

### 2.3. Euthanasia and Sample Preparation

At the time of scarification, blood was collected by heart puncture into plasma-EDTA and serum tubes (Sarstedt, Nümbrecht, Germany) under deep isoflurane anaesthesia (Forane, Abbott, Austria). After centrifugation at 2000× *g* for 12 min at room temperature, plasma and serum samples were aliquoted and stored at −80°C until batched analysis. Immediately after blood collection, the following organs were explanted and snap frozen in liquid nitrogen: liver, skeletal muscle, heart, aorta, large intestine, spleen, kidney, brain, lung, visceral fat. Subsequently, all tissue samples were stored together deep-frozen at −80°C until analysis. Exclusion criteria were the development of illnesses or tumours during the intervention period.

### 2.4. Analysis of Relative Telomere Length (RTL) in PBMCs and Solid Organs

After diluting 100 µL of whole blood with 100 µL of dH_2_O, DNA was isolated with the MagNA Pure LC instrument (Roche, Austria) using the Total Nucleic Isolation Kit (Roche, Austria). Subsequently, relative telomere length (RTL) of peripheral blood mononuclear cells (PBMCs) was measured by quantitative real-time PCR (qPCR) using a protocol developed by Cawthon [65]. This assay quantifies the ratio of average TL (T) to glyceraldehyde-3-phosphate dehydrogenase (GAPDH) as single copy reference gene (S). The single copy gene is used as amplification control for each sample and to determine the number of genome copies per sample. All qPCR analyses were performed on a Thermocycler CFX384 TouchTM (Biorad, Feldkirchen, Germany) instrument using the following primers:

1. Telomere For: 5′-CGGTTTGTTTGGGTTTGGGTTTGGGTTTGGGTTTGGGTT-3′;
2. Telomere Rev: 3′-GGCTTGCCTTACCCTTACCCTTACCCTTACCCTTACCCT-5′;
3. GAPDH For: 5′-CACCTAGACAAGGATGCAGAG-3′;
4. GAPDH Rev: 3′-GCATGACTGGAGGAATCACA-5′.

All primers have been purchased from Eurofins Genomics, Austria. Each run included a standard curve made by dilutions of isolated and pooled rat DNA from 21 different blood samples, to determine the quantity of the targeted templates. RTL has been calculated as the ratio of telomere quantity to single copy reference gene quantity (T/S ratio).

RTL in solid organs was analysed with the same method described before. For this purpose, approximately 10 mg of tissue were homogenised in 300 µL Magna Pure Lysis Buffer (Roche, Wien, Austria) using the MagnaLyser (Roche, Wien, Austria). Subsequently, the DNA was isolated and quantified using the same procedure as for blood leucocytes.

### 2.5. The mRNA Expression Analyses in Blood Cells and Solid Organs

TERT, TERF-1, and TERF-2 gene expression was analysed in RNA extracts of all solid organs. As blood leucocytes were used up for the measurement of RTL, they were not available for mRNA expression analyses. Therefore, mRNA expression in spleen was used as reference because the organ belongs to the lymphatic system and is rich in leucocytes. From each organ, 10 mg of tissue were homogenised in 300 µL Magna Pure Lysis Buffer (Roche, Wien, Austria) using the MagnaLyser (Roche, Wien, Austria). RNA was extracted from these homogenates with the Total Nucleic Isolation Kit (Roche, Wien, Austria) on a MagNA Pure LC instrument (Roche, Wien, Austria). Subsequently, the mRNA in these extracts was transcribed into cDNA using the QuantiTect Reverse Transcription kit (Qiagen, Hilden, Germany). Finally, mRNA expression of TERT, TERF-1, and TERF-2 was analysed by qPCR with TaqMan probes (Life Technologies dba Invitrogen, Waltham, MA, USA). The expression of each target gene expression was calculated with the ΔΔCT method using β-actin as reference gene. The sequences of the probes used were as follows:

5. Β-actin: 5′-CTTCCTTCCTGGGTATGGAATCCTG-3′;
6. Tert: 5′-ATCGAGCAGAGCATCTCCATGAATG-3′;
7. Terf-1: 5′-AAAACAGACATGGCTTTGGGAAGAA-3′;
8. Terf-2: 5′-GAGAAAATTTAGACTGTTCCTTTGA-3′.

### 2.6. Statistical Analyses

Results are shown as mean ± standard deviations (SD). Qualitative variables such as tumor abundance and type were assessed with the Fisher’s exact test or the Chi-squared test. Group differences were assessed using the two-tailed Student’s *t* test for dependent or independent samples or the Mann–Whitney U test depending on the distribution of the data. Group comparisons with three or more groups were analysed using the two-way ANOVA or the Kruskall–Wallis test for independent samples. Correlations between variables were determined by linear regression analysis according to Pearson (r, Pearson Correlation coefficient; p, univariate ANOVA). Data were plotted using Python programming language with Jupyter Notebook within the data science package Anaconda3 for Windows. IBM SPSS v. 26 for Windows was used for explorative data analysis and a level of acceptance of the null hypothesis was set at *p* = 0.05.

## 3. Results

### 3.1. Characterization of the Animal Model

From the 96 rats, 6 were excluded prior to the end of the study due to general health issues. A total 18 animals developed benign tumours and, thus, were excluded from the final analysis. Tumours were more frequent in animals on HFD rather than on ND (16 vs. 2 rats, *p* = 1.289 × 10^−4^). The tumours in the HFD animals were of heterogeneous nature compared to the ND group (*p <* 0.001), as masses were found in breasts, ovaries, and abdomen of obese animals. Regular exercise did not significantly change tumor incidence in both diet groups (coND vs. exeND, *p* = 0.975; coHFD vs. exeHFD, *p* = 0.347) nor tumor diversity in the HFD group (*p* = 0.197). After exclusion of dropouts, 72 eligible animals were included in the final statistical analyses. 

**Figure 1:**
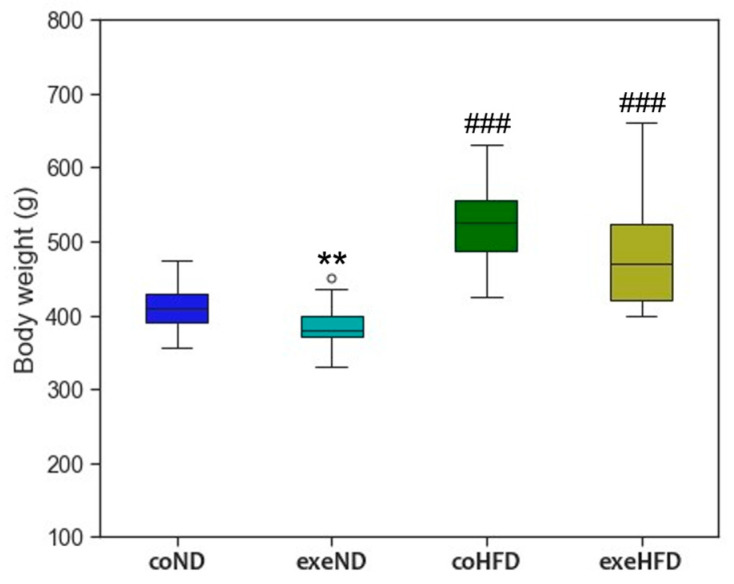
Box and Whisker plot of the body weight at the end of the 10 months study period. Outliers are shown as white circles above the box plots. The two-tailed Student’s *t*-test was used for group comparison of independent samples. ** *p* < 0.01 compared to appropriate sedentary control group; *^###^ p* < 0.001 compared to appropriate normal diet control group.

At study end, the animals in the two HFD groups had a significantly higher body weight than those in the ND groups (Figure 1). Median body weight between ND and HFD differed by 115 g in sedentary animals and by 90 g in exercising animals. In line with this finding, also the weight of individual organs and tissues, such as heart, liver, and visceral fat, was significantly higher in HFD animals (Table 1).

The exercise protocol was well tolerated by the animals of both diet groups. Body weight of exeND animals was significantly lower than that of coND animals (*p* < 0.01), whereas coHFD and exeHFD animals showed no difference. In the factorial ANOVA, the main effects of diet and exercise on body weight were significant with F (1, 67) = 80.92, *p* = 3.89 ×10^−13^, and F (1, 67) = 8.29, *p* = 0.005, respectively. There was no significant interaction between diet and exercise, F (1, 67) = 0.138, *p* = 0.712. Regular exercise induced a higher organ weight of heart and liver in HFD animals, but not in ND animals (Table 1).

### 3.2. Influence of HFD on RTL and the Expression of Telomere-Regulating Genes in Different Tissues 

**Figure 2:**
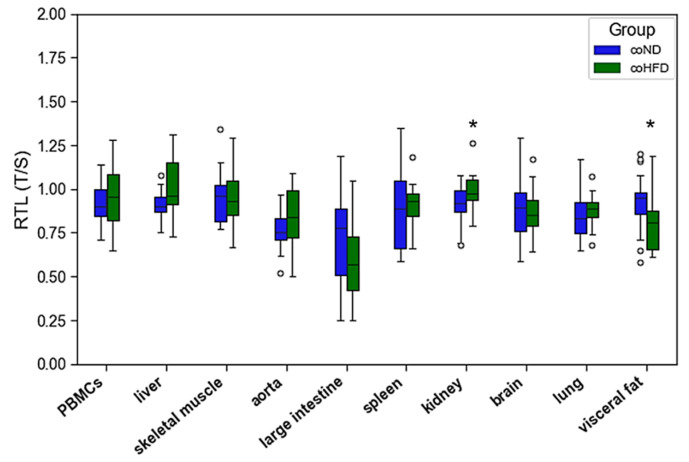
Distribution of RTL in PBMCs and nine solid organs isolated from lean (coND) and obese rats (coHFD). Outliers are shown as white circles above the box plots. RTL is expressed as ratio of average telomere length to the reference gene GAPDH. The two-tailed Student’s *t*-test or the Mann–Whitney U-test were used for group comparison of independent samples. * *p* < 0.05 vs. coND.

When compared to ND, 10 months of HFD consumption had no systematic effect on RTL across different organs. In visceral fat RTL was significantly lower in coHFD animals than in coND animals. In contrast, renal RTL was slightly higher in coHFD than in coND animals. All other solid tissues and PBMCs showed comparable RTL between the two diet groups. (Figure 2).

**Figure 3:**
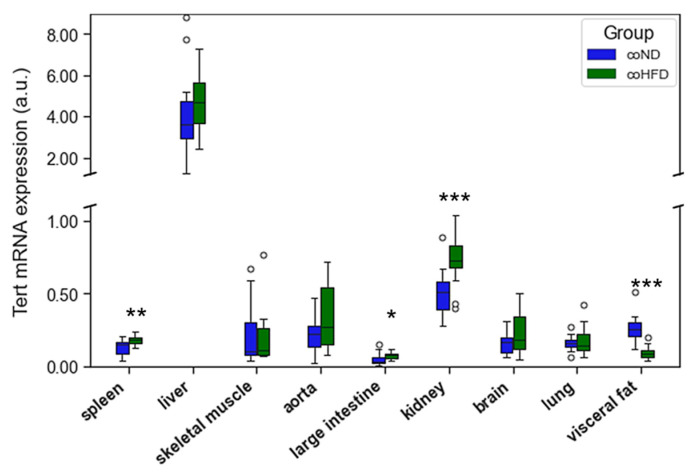
Differences in the TERT gene expression isolated from different solid organs of lean (coND) and obese rats (coHFD). Outliers are shown as white circles above the box plots. TERT mRNA expression is shown in arbitrary units. The two-tailed Student’s *t*-test or the Mann–Whitney U-test were used for group comparison of independent samples. * *p <* 0.05; ** *p <* 0.01; *** *p <* 0.001 vs. coND.

TERT mRNA expression varied substantially between tissues with highest expression levels in liver and kidney. The consumption of HFD did not result in a systematic difference of TERT mRNA expression across different organs (Figure 3). Spleen, large intestine, and kidney showed higher TERT mRNA expression levels in coHFD than in coND animals, whereas in visceral fat a lower TERT mRNA expression was observed.

**Figure 4:**
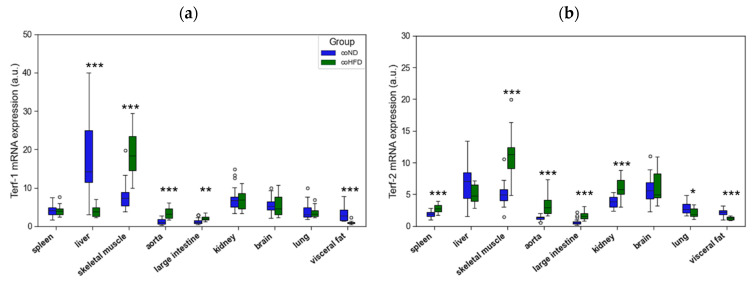
Differences in the gene expression of (**a**) TERF-1, and (**b**) TERF-2 isolated from different solid organs of lean (coND) and obese rats (coHFD). Outliers are shown as white circles above the box plots. TERF-1, and TERF-2 mRNA expression is shown in arbitrary units. The two-tailed Student’s *t*-test or the Mann–Whitney U-test were used for group comparison of independent samples. * *p* < 0.05; ** *p* < 0.01; *** *p* < 0.001 vs. conD.

**Figure 5:**
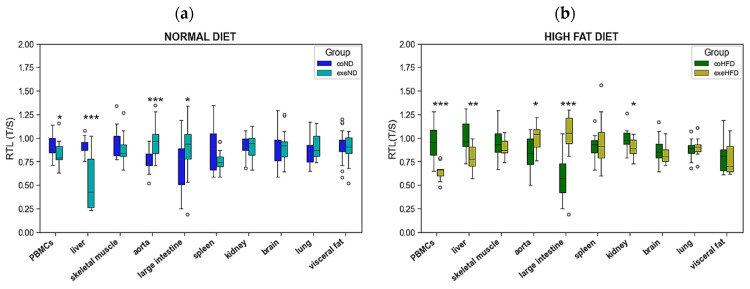
Comparison of RTL in PBMCs and nine solid organs isolated from exercising and sedentary SD rats that received normal diet or HFD for 10 months. (**a**) sedentary (coND) vs. exercising (exeND) animals on ND, (**b**) sedentary (coHFD) vs. exercising (exeHFD) animals on HFD. Outliers are shown as white circles above the box plots. RTL is expressed as ratio of average telomere length to the reference gene GAPDH. The two-tailed Student’s t-test or the Mann–Whitney U-test were used for group comparison of independent samples. * *p* < 0.05, ** *p* < 0.01, *** *p* < 0.001 vs. respective sedentary controls.

Similar to TERT, also mRNA expression of the two shelterins, TERF-1 and TERF-2, varied substantially between tissues. The consumption of HFD upregulated TERF-1 and TERF-2 mRNA expression in 3 (Figure 4a) and 5 (Figure 4b) out of nine tissues, respectively. In contrast, a reduced mRNA expression of both shelterins was seen in only one and two tissues, respectively. A simultaneous upregulation of TERF-1 and TERF-2 in coHFD animals was found in skeletal muscle, aorta, and large intestine. In contrast, visceral fat showed a lower mRNA expression of both shelterins in these animals. Furthermore, TERF-1 was markedly reduced in the liver of coHFD rats, whereas TERF-2 was increased in spleen and kidney but decreased in lung tissue.

Combining all the differences described before in an effect matrix, it becomes apparent that only in visceral fat HFD consistently reduces RTL and the mRNA expression of telomere-regulating genes (Figure S1). Instead, in four out of nine tissues mRNA expression of one or more telomere-regulating genes was increased without a change in RTL.

### 3.3. Influence of Exercise on RTL and the Expression of Telomere-Regulating Genes in Different Tissues 

Ten months of regular treadmill running had heterogeneous effects on RTL in different tissues with significantly longer telomeres in aorta and large intestine tissue, but shorter telomeres in PBMCs and liver RTL (Figure 5). In all other tissues, RTL did not significantly differ between sedentary and exercising animals. Of note, the simultaneous administration of HFD did not substantially change this pattern.

**Figure 6:**
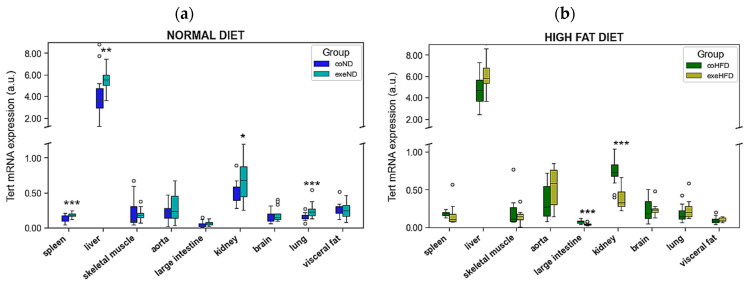
Comparison of TERT expression in nine solid organs from exercising and sedentary SD rats that received either normal diet or HFD for 10 months. (**a**) sedentary lean animals (coND) vs. exercising lean animals (exeND), (**b**) sedentary obese animals (coHFD) vs. exercising obese animals (exeHFD). Outliers are shown as white circles above the box plots. TERT mRNA expression is shown in arbitrary units. The two-tailed Student’s *t*-test or the Mann–Whitney U-test were used for group comparison of independent samples. * *p* < 0.05; ** *p* < 0.01; *** *p* < 0.001 vs. respective sedentary controls.

Exercise had vastly different effects on mRNA expression of TERT, TERF-1 and TERF-2 in different tissues. In some tissues, but not all, HFD altered the exercise-induced effects observed in ND animals. TERT mRNA expression was increased in spleen, liver, kidney, and lung of exeND animals compared to coND animals (Figure 6a). Conversely, in exeHFD animals, TERT expression in large intestine and kidney was significantly lower than in coHFD, whereas spleen, liver and lung showed no differences (Figure 6b).

**Figure 7:**
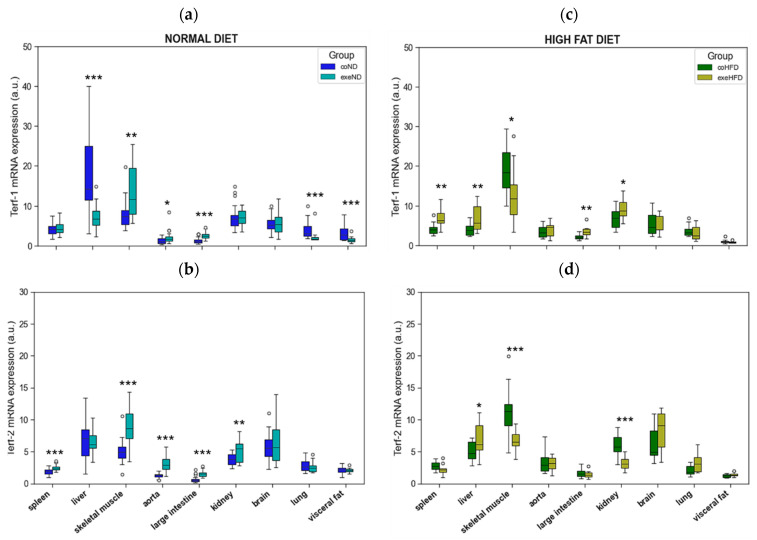
Comparison of TERF-1 and TERF-2 expression in nine solid organs from exercising and sedentary SD rats that received either normal diet or HFD for 10 months. (**a**) TERF-1 in sedentary lean animals (coND) vs. exercising lean animals (exeND), (**b**) TERF-2 in sedentary lean animals (coND) vs. exercising lean animals (exeND) (**c**) TERF-1 in sedentary obese animals (coHFD) vs. exercising obese animals (exeHFD), (**d**) TERF-2 in sedentary obese animals (coHFD) vs. exercising obese animals (exeHFD). Outliers are shown as white circles above the box plots. TERF-1 and TERF-2 mRNA expression is shown in arbitrary units. The two-tailed Student’s *t*-test or the Mann–Whitney U-test were used for group comparison of independent samples. * *p* < 0.05; ** *p* < 0.01; *** *p* < 0.001 vs. respective sedentary controls.

TERF-1 mRNA expression was significantly reduced in liver, lung, and visceral fat, but increased in skeletal muscle, aorta, and large intestine of exeND rats when compared to coND animals (Figure 7a). In exeHFD animals instead, TERF-1 mRNA expression was increased in spleen, liver, large intestine, and kidney, but reduced in skeletal muscle when compared to coHFD animals (Figure 7c).

TERF-2 mRNA expression was significantly increased in five out of nine solid organs of exeND animals when compared to coND rats (Figure 7b), namely spleen, skeletal muscle, aorta, large intestine, and kidney. In exercising obese animals instead, TERF-2 mRNA expression was profoundly different with a higher expression level in liver, but reduced expression levels in skeletal muscle and kidney in exeHFD rats when compared to coHFD animals (Figure 7d).

Summarizing all the results from sedentary and exercising lean animals in the effect matrix (left column), it becomes clear that only in aorta and large intestine an increase in one or more telomere-regulating genes was associated with an increase in RTL (Figure S1). All other differences in mRNA expression of telomere-regulating genes were unrelated to RTL. Similar to exercise, also HFD failed to induce systematic effects on RTL and telomere-regulating genes. Only in kidney and visceral fat of obese sedentary animals, did RTL and telomere-regulating genes show changes directed in the same way. However, the changes in both tissues pointed in opposite directions. An interaction between HFD and exercise was only observed in kidneys, where exercising obese rats exhibited a similar RTL to sedentary lean controls (Figure S1). Additionally, both exercising groups show reductions in hepatic RTL, but inconsistent changes in the hepatic expression of telomere-regulating genes.

### 3.4. Correlation Analysis

**Figure 8:**
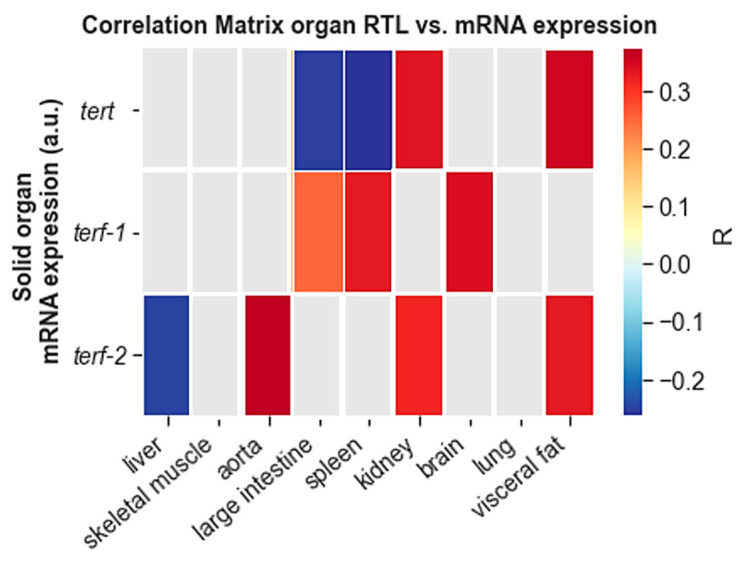
Correlation matrix for RTL and telomere-regulating genes in 9 solid tissue types. The colour bar on the right side of the figure shows the Pearson correlation coefficient as R. Only significant correlations are shown in colour (*p* ≤ 0.05). Grey boxes indicate the lack of significant correlation.

To further explore our hypothesis that lifestyle factors can modify RTL through regulatory effects on the expression of telomere-regulating genes, we performed correlation analyses that included the animals from all four groups. Figure 8 illustrates that RTL was not consistently correlated to any of the telomere-regulating genes. For example, in kidney (*R* = 0.337; *p* = 0.004) and visceral fat (*R* = 0.337; *p =* 0.004) RTL and TERT mRNA expression were positively correlated, whereas large intestine (*R* = −0.252; *p =* 0.036) and spleen (*R* = −0.263; *p* = 0.028) showed the opposite. Likewise, RTL and TERF-2 were positively correlated in aorta (*R =* 0.373; *p =* 0.002), kidney (*R* = 0.318; *p* = 0.007) and visceral fat (*R =* 0.332; *p =* 0.007), but negatively correlated in liver (*R =* −0.247; *p* = 0.053).

## 4. Discussion

Here, we show for the first time that neither regular exercise nor the consumption of HFD have a systematic effect on RTL in solid tissues and PBMCs of SD rats. In fact, most tissues had comparable RTL in the respective intervention and control groups. Additionally, dual stimulation by feeding HFD to exercising animals did not change this result. Nevertheless, some tissues exhibited significantly higher RTL after 10 months of HFD (kidney) or exercise (aorta and small intestine), whereas other tissues showed reduced RTL upon HFD (visceral fat) or exercise (PBMCs and liver). These differences were not accompanied by a consistent mRNA expression pattern of the respective telomere-regulating genes *tert*, *terf-1* and *terf-2*. Therefore, the present results do not support the hypothesis that regular moderate endurance exercise or prolonged exposure to a diet rich in saturated lipids influence RTL through the expression of telomerase and shelterins.

The comprehensive mapping of RTL and related telomere-regulating genes after long-term exposure to HFD and exercise significantly expands existing knowledge on the influence of modifiable lifestyle factors on age-related telomere shortening. Previous in vivo studies have mostly focused on RTL in specific cells or tissue types, such as PBMCs or myocardium [16,17,21,60]. The results are rather inconsistent. Ludlow et al. showed in wild type derived short telomere mice (CAST/Ei) that 1 year of voluntary running in a running wheel preserved TL in myocardium and liver, but not in skeletal muscle [60]. Similar to the present study, these effects were not accompanied by consistent alterations of telomere-regulating genes that would explain these effects. In contrast, after 3 weeks of voluntary wheel running, Werner et al. reported an upregulation of telomerase activity (TA) in murine aorta and PBMCs, and an increased aortic gene expression of TERF-2. Additionally, senescence-associated genes, such as Chk2, p53, and p21, were lowered in the aorta of exercising animals. However, the increased expression of these telomere-regulating genes did not result in a significant difference of aortic TL after 6 months of exercise when compared to inactive controls [17]. The TL results reported by the two studies of Ludlow et al. and Werner et al. are not in line with our findings, where 10 months of regular moderate running exercise reduced RTL in PBMCs and liver, whereas aorta and large intestine showed a significant increase.

The inconsistencies between existing exercise studies in animals may, at least partly, be explained by differences in the animal models used. Our results were obtained in SD rats, whereas previous studies worked with CAST/Ei [60] and C57/Bl6 mice [17]. Additionally, the duration of exercise varied amongst existing studies between a few weeks and one year, which further limits comparability. In addition, a greater group size with 22 coND and 22 exeND animals provides robustness to our results. The studies from Ludlow et al. and Werner et al. were performed with no more than 10 animals per group, which limits statistical power and leaves more room for random effects. A major strength of the present study is strict standardization of the exercise intervention, which consisted in forced treadmill running for 30 min at fixed speed on 5 consecutive days per week. The efficacy of this intervention is evidenced by a significantly lower body weight at the time of scarification. In contrast, most previous studies used voluntary wheel running, which is not standardized.

Similar to the exercise studies discussed before, mouse models of obesity and metabolic syndrome also failed to show accelerated telomere shortening despite an upregulation of Chk2, p53, and p21 [22,23]. For example, feeding mice for 60 weeks with a high-fat/high-sucrose diet induced obesity and metabolic dysfunction, but did not accelerate LTL shortening [23]. With advancing obesity, the animals were physically less active, which should have amplified potentially adverse effects of obesity. Additionally, in genetically modified rats with metabolic syndrome, Takahashi et al. showed comparable myocardial TL than in wild-type controls [22]. At the same time, telomerase expression and TA were upregulated together with the senescence-associated genes Chk2, p53, and p21. These results are in line with our present study, showing similar RTL in PBMCs, liver, aorta, and skeletal muscle after 10 months of HFD or ND. Additionally, large intestine, spleen, brain, and lung showed comparable RTL in the two groups. In our model neither exercise nor HFD induced a consistent expression pattern of telomere-regulating genes, namely *tert*, *terf-1* and *terf-2*. Only kidney and visceral fat showed significant differences in RTL, but in opposite directions. Similar differences were detected for the expression of *tert* and *terf-2*. However, the relevance of these effects is questionable as TERT, TERF-1 and TERF-2 were also altered in several other tissues of HFD animals without affecting the respective RTL. Furthermore, most existing studies reported effects of exercise and obesity on telomerase and shelterins, but often this was not associated with changes in RTL. In line with existing data, correlation analyses in the present study showed inconsistent correlations between RTL and mRNA expression levels of the three telomere-regulating genes. Altogether, these results question the pathophysiological relevance of such observations.

A unique aspect of the present study is the combination of exercise and HFD. In modern societies, people often try to compensate adverse nutritional habits with exercise, but the efficacy of this approach is not well documented. Our results show that such an approach does produce a different outcome than exercise alone. Specifically, RTL was lower in PBMCs, liver and kidney of exercising animals on HFD, but higher in aorta and large intestine. Despite a comparable pattern of RTL in the different organs of exercising animals on normal diet and HFD, incongruent results were registered for the mRNA expression of *tert*, *terf-1* and *terf-2*. For example, *terf-1* and *terf-2* were both increased in skeletal muscle of exercising animals on ND but decreased in exercising animals on HFD. However, both groups showed comparable RTL in this tissue. In line with this argument, also correlation analyses that included all 72 animals did not show consistent correlations between RTL and the expression level of telomerase or shelterins. For example, an inverse correlation between RTL and *tert* was seen in spleen and large intestine, whereas kidney and visceral fat showed the opposite. In all other tissues both parameters were not correlated.

This present animal study does not support the results from human studies showing a reduced LTL in obese people [39,40] and a preservation of TL upon regular endurance exercise [17]. Although some studies do not support an inverse relationship between TL and obesity [41,42,43], a recent meta-analysis calculated a significantly lower LTL in obese individuals than in normal-weight individuals [49]. Moreover, LTL was inversely correlated with BMI, body fat content, waist circumference, waist-to-hip ratio, and nuchal fat thickness. However, the observational character of the studies included impedes any conclusion towards causality. Additional insights can be gained from longitudinal observation studies that assessed LTL in obese patients before and after bariatric surgery [66]. Available results indicate an improvement in LTL after >2 years, probably due to an improvement in inflammation and oxidative stress. However, only a small number such studies has been published, with rather heterogenous design and outcome. Human studies that investigated LTL in exercising and sedentary individuals are also inconsistent. Several observational studies have shown higher LTL in exercising individuals of different age groups and activity levels [15,17,18,57,58]. Additional support from prospective observation and intervention studies is strongly limited. Soares-Miranda L et al. performed serial blood collections over a 5-year period in 582 older US adults and found no significant association between physical activity, physical performance, and LTL [59]. In contrast, Werner et al. reported an increase in LTL, TA, and TERF-2 expression after 6 months of aerobic endurance training or high intensity training, which was not seen in controls [17,18].

A general downside of existing human studies is the limitation of TL analyses to blood leucocytes, which impedes conclusions about TL in solid tissues of obese and lean individuals. However, previous results from our group have shown that LTL does not provide reliable information on TL in other tissues [67]. While RTL in some tissues, exhibit a positive correlation with LTL, others show the opposite. Additionally, RTL in young and aged SD rats did not systematically change.

The present results should be interpreted with caution keeping in mind the strengths and limitation of this study. A rather large number of animals per group and a strictly standardized exercise intervention provide robustness to the results. In addition, the intervention period was quite long. However, results from Werner et al. suggest, that up to 18 months may be needed to observe a significant reduction in TL [16]. SD rats have an average life expectancy of 2 years so that our animals were sacrificed at advanced adult age, but they cannot be regarded old. The exercise protocol applied was rather moderate and a more intensive regimen might have produced different results. However, with this protocol we aimed to mimic a common recreational activity pattern in adults. Energy intake and energy expenditure may have varied between individual animals and different groups. The lacking information on both factors adds some uncertainty to the interpretation of our results. Another important limitation is the RT-qPCR method that has been used for the measurement of RTL. This method gives an average TL across all cells and chromosomes but does not provide information on the percentage of very short and long telomeres. There is some evidence that the percentage of very short telomeres rather than average TL is associated with aging and age-related disease [68]. However, determination of the shortest telomeres requires highly sophisticated and cumbersome methods, such as Telomere Shortest Length Assay (TeSLA) [69]. In addition, these methods are difficult to standardize and not suitable for high throughput analysis. As we had planned to analyze more than 1000 samples, these assays were deemed not feasible for our purpose. Lastly, telomere-regulating genes were only analysed by mRNA expression, but not at protein level. Although mRNA expression and protein analyses may give discordant results, we do not feel that this limits the overall meaning of our results. The absence of systemic effects on RTL in PBMCs and solid tissues and the highly inconsistent mRNA expression pattern of telomerase and shelterins limit the potential scope of these factors as relevant mediators of telomere effects induced by exercise and diet.

## 5. Conclusions

In summary, the present in vivo study does not provide evidence that modifiable lifestyle factors, such as obesity and exercise, have significant systemic effects on telomere shortening and the expression of telomere-regulating genes. Additionally, exercise and HFD do not show significant interaction. Any lifestyle-related effect on RTL and telomere-regulating genes in one tissue type does not allow conclusions on other tissues or cell types. Future research should address the impact of exercise and diet on the shortest telomeres and explore their role for aging and degenerative disease. Moreover, future studies on the effects of lifestyle factors on telomere length and telomere function should focus on advanced adult age, where degenerative disease most frequently occurs.

## Figures and Tables

**Table 1:** Organ weight in female SD rats after 10 months of treadmill exercise.

Organs	Measurement	ND		HFD	
		Sedentary *n* = 22	Exercising *n* = 22	Sedentary *n* = 16	Exercising *n* = 12
heart	average weight	1.31 ± 0.21	1.24 ± 0.11	1.40 ± 0.14	1.46 ± 0.19 ^###^
	normalized weight	0.28 ± 0.04	0.27 ± 0.03	0.30 ± 0.03	0.31 ± 0.03 ^###^
spleen	average weight	0.98 ± 0.16	0.97 ± 0.15	1.20 ± 0.16 ^###^	1.18 ± 0.23 ^##^
	normalized weight	0.21 ± 0.03	0.21 ± 0.03	0.24 ± 0.07	0.25 ± 0.04 ^###^
liver	average weight	12.53 ± 1.72	12.50 ± 1.80	14.03 ± 2.36 ^#^	15.16 ± 4.40 ^#^
	normalized weight	2.67 ± 0.31	2.56 ± 0.68	2.98 ± 0.52 ^#^	3.28 ± 0.89 ^#^
visceral fat	average weight	13.20 ± 5.26	10.46 ± 4.48	40.13 ± 12.81 ^###^	39.46 ± 23.20 ^###^
	normalized weight	0.03 ± 0.01	0.03 ± 0.01	0.08 ± 0.018 ^###^	0.07 ± 0.03 ^###^

Organ weight is given in grams. The weights of heart, spleen, and liver were normalized to total tibia length (cm), while visceral fat weight was normalized to body weight (g). Data are presented as mean ± SD; ^#^ *p* < 0.05, ^##^ *p* < 0.01, ^###^ *p* < 0.001 compared to the appropriate normal diet control group with the two-tailed Student’s *t*-test for independent samples.

## Data Availability

All data underlying the findings reported in this manuscript are provided as part of the article. The raw data that are not already presented in the figures are available from the corresponding author upon reasonable request.

## Supplementary Materials

The following supporting information can be downloaded at: www.mdpi.com/article/10.3390/cells11101605/s1, Figure S1: Effects matrix that visualizes the effects of exercise, HFD, and the interaction of both lifestyle factors on RTL and the mRNA expression of telomere-regulating genes.
