# Supplementary material for: Influences of Long-Term Exercise and High-Fat Diet on Age-Related Telomere Shortening in Rats
Source: Cells. 2022 May 10;11(10):1605. doi: 10.3390/cells11101605 (PMC9139508; doi:10.3390/cells11101605)
Supplement: Supplementary file 1 [file cells-11-01605-s001.zip › cells-1707154-supplementary.pdf]

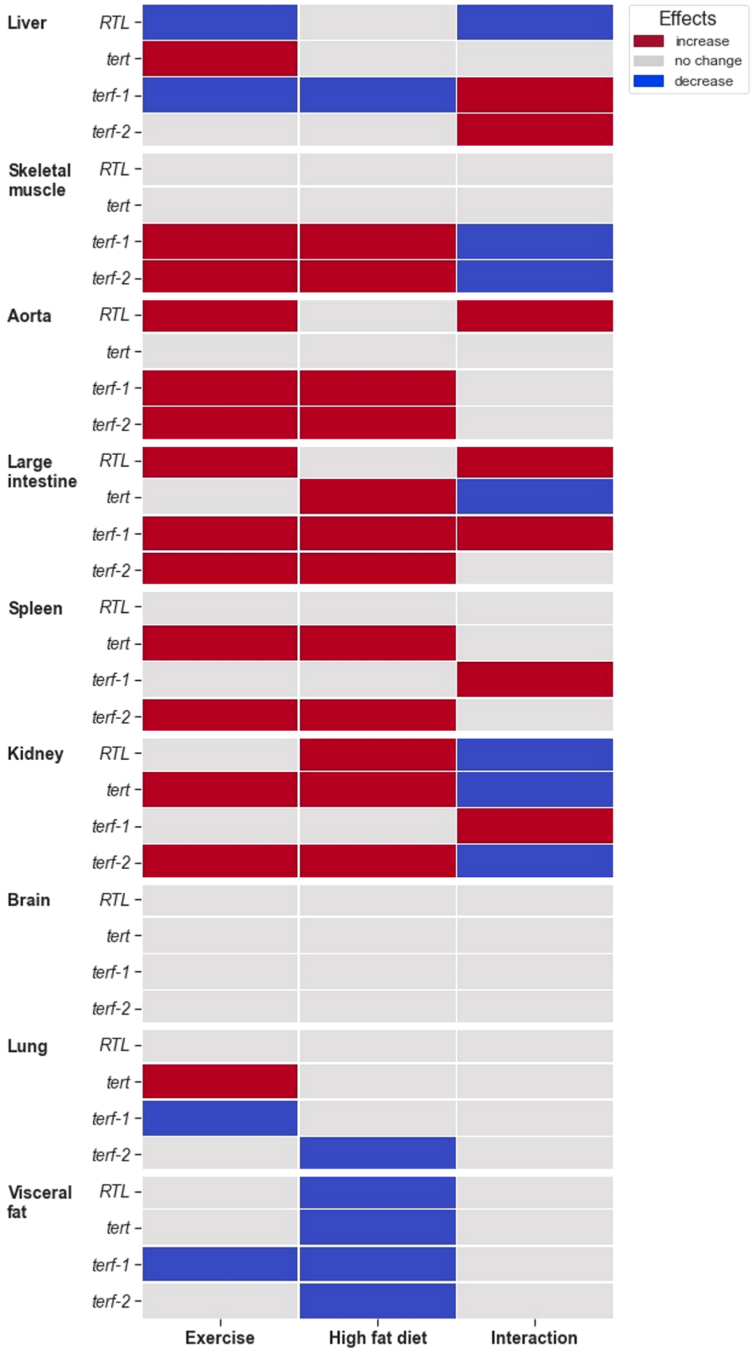

Figure S1: Effects matrix that visualizes the effects of exercise, HFD, and the interaction of both lifestyle factors on RTL and the mRNA expression of telomere-regulating genes. The two-tailed Student's t-test was used for independent samples' comparison. Significant effects are shown in color and refer to the respective control group ($p \leq 0.05$). Grey boxes indicate the lack of a significant effect.
